# Implementing post-normal science with or for EU policy actors: using quantitative story-telling

**DOI:** 10.1007/s11625-022-01265-1

**Published:** 2023-01-20

**Authors:** Kirsty L. Blackstock, K. A. Waylen, K. B. Matthews, A. Juarez-Bourke, D. G. Miller, A. Hague, D. H. Wardell-Johnson, M. Giampietro

**Affiliations:** 1 Social, Economic and Geographical Sciences, James Hutton Institute, Craigiebuckler, Aberdeen, AB15 8QH Scotland, UK; 2 Information and Computational Sciences, James Hutton Institute, Craigiebuckler, Aberdeen, Scotland, UK; 3 Institute of Environmental Science and Technology (ICTA), Universitat Autònoma de Barcelona, Bellaterra, Barcelona, Spain; 4 ICREA, Pg. Lluís Companys 23, Barcelona, Spain

**Keywords:** Quantitative story-telling, Post-normal science, Science–policy interactions, European Union, Agriculture

## Abstract

There is increasing recognition of the wicked nature of the intertwined climate, biodiversity and economic crises, and the need for adaptive, multi-scale approaches to understanding the complexity of both the problems and potential responses. Most science underpinning policy responses to sustainability issues, however, remains overtly apolitical and focussed on technical innovation; at odds with a critical body of literatures insisting on the recognition of systemic problem framing when supporting policy processes. This paper documents the experience of implementing a mixed method approach called quantitative story-telling (QST) to policy analysis that explicitly recognises this normative dimension, as the methodology is part of a post-normal science (PNS) toolkit. The authors reflect on what was learnt when considering how QST fared as a tool for science–policy interaction, working with European Union (EU) level policy actors interested in sustainable agriculture and sustainable development goal 2. These goals—also known as UN Agenda 2030—are the latest institutionalisation of the pursuit of sustainable development and the EU has positioned itself as taking a lead in its implementation. Thus, the paper illustrates our experience of using PNS as an approach to science policy interfaces in a strategic policy context; and illustrates how the challenges identified in the science–policy literature are amplified when working across multiple policy domains and taking a complex systems approach. Our discussion on lessons learnt may be of interest to researchers seeking to work with policy-makers on complex sustainability issues.

## Introduction

The publication of the UN 2030 agenda and its 17 sustainable development goals (SDGs) renewed rhetorical commitment to sustainability. The Agenda and SDGs promote shared responsibility between the global North and South; and the involvement of societal actors from the public, private and civic domains (Simon and Schiemer 2015). However, policy processes associated with the SDGs have been critiqued for retaining a managerial approach focussed on technical innovations (Koff and Maganda 2016). Failing to take a systemic approach to delivery of the SDGs (Norström et al. 2014) and giving insufficient attention to political choices and power imbalances perpetuates inequitable and unsustainable outcomes (Blühdorn 2011).

Understanding sustainable development as a ‘wicked problem’ (Termeer et al. 2015) highlights the political and subjective nature of how sustainability problems and solutions are framed. Although scientific knowledge plays a critical role in the understanding of interconnected sustainability challenges and responses (Clark and Dickson 2003), science is not neutral and separate to these wicked problems. Facilitating appropriate use of science (and other) knowledges for sustainability is at the heart of science–policy interfaces (SPIs) literature.

Decades of scholarship on science–policy interfaces or SPIs (e.g. van den Hove 2007) and the role of science in decision-making (e.g. Jasanoff and Wynne 1998) plus current recommendations on transdisciplinary research (Bergmann et al. 2021) provide a variety of insights and recommendations for improving scientific knowledge use in policy processes. In particular, there is a need to better acknowledge political factors inherent to science knowledge (Wesselink et al. 2013); better value and reflect different knowledge systems (Cornell et al. 2013; e.g. Norström et al. 2020); and the multiple factors shaping policy processes (Cairney 2012); and recognise limits to our certainty and control of socio-ecological systems (Partelow 2015; Miller et al. 2013).

In practice, the SPI literature draws attention to the need, at minimum, to co-produce research agendas (Sutherland et al. 2012) and ideally, to ensure that scientific knowledge is produced, interpreted and used with the policy actors for whom the science was designed to support. Instead of knowledge transfer, a dialogue is needed to comprehend and shape how knowledge is needed, understood, and used (Young et al. 2014). However, these ideas are not always reflected by practice; how to foster new co-produced approaches remains unclear (Cundill et al. 2019), and aspirations for transformative sustainability science are often disappointed (Jagannathan et al. 2020). These disappointments may be due to the failure to take a politically informed approach to knowledge co-production in SPIs (Maas et al. 2022).

This suggests that a ‘post-normal science’ perspective on sustainability might be useful. Post-normal science (PNS) is suggested for when the "facts [are] uncertain, values in dispute, stakes high and decisions urgent" (Funtowicz and Ravetz 1993). It is therefore relevant to many contemporary sustainability challenges. PNS is a normative philosophy not a precise methodological prescription (Turnpenny et al. 2011); providing room for heterodox interpretations. Despite the potential application of PNS approaches to policy support, there are very few examples of PNS being used with EU policy actors in transdisciplinary teams. Instead, PNS research focus tends to be whether practices are post-normal (Karpińska 2018; Rose 2018; Ainscough et al. 2018); focussed on a specific controversy (Palliser and Dodson 2017); or working with specific local actors (Tsukahara 2017). This paper analyses the use of PNS with policy actors within EU institutions and considers how PNS informs the science–policy interface.

To understand how PNS works to support science–policy interfaces, we consider attributes seen as relevant to both PNS and SPIs. First, fundamental to effective SPIs are concepts of Credibility, Relevance (salience) and Legitimacy (CRELE) (Cash et al. 2002) to ensure that scientific findings are judged by policy-makers as relevant and timely, based on robust methodologies using transparent and inclusive processes. More recently, Sarkki et al. (2015) added ‘iterativity’ to credibility, relevance and legitimacy, to recognise the dynamics involved in these policy–science exchanges (van Hulst and Yanow 2016). PNS scholars use the TRUST (transparency, robustness, uncertainty, sustainability and transdisciplinary) mnemonic (Kønig et al. 2017).

Transparency and robustness are shared with CRELE whilst a focus on sustainability is one manifestation of relevance. However, uncertainty, particularly ontological uncertainty, arises for non-equivalent perspectives on the same phenomena, and emphasises the importance of exploring multiple problem frames rather than assuming problems are agreed or solutions straightforward. This is because it is problematic to impose structured solutions on wicked problems that may not recognise the wider systemic issues (Hisschemöller and Hoppe 2001). With respect to SPIs, a PNS approach implies that policy actors should work with scientists to ‘find the problem’ (Hoppe 2018) before they can discuss solutions to avoid screening out difficult systemic considerations.

Trans-disciplinarity highlights how knowledge is held by many actors beyond formally qualified scientific experts, so problem structuring requires the input of ‘transdisciplinary teams’ of varied societal actors (Alonso-Yanez et al. 2019). Therefore a PNS approach to SPIs requires policy actors to be active knowledge producers as well as knowledge users. Furthermore, to tackle wicked problems, such as sustainable development, SPI actors need to be responsive and interested in difficult political challenges (Termeer et al. 2015). Many papers on SPIs highlight the relational and longitudinal demands of the process on individuals, e.g. Sarkki et al. (2019) and Bergmann et al. (2021).

These individual capacities are enacted within a wider context (Sarkki et al. 2019), in this case governance processes, that balance steering and coordination by the EU with the autonomy of member states (Schout and Jordan 2005; Rüffin 2020). These processes include the European Commission, its agencies and the political arenas of the Parliament, Council of Europe and Council of Ministers. Policy development, implementation and evaluation processes are steered by collective discourses and behaviours (Candel et al. 2014; Voelker et al. 2019; Gossling et al. 2016). Therefore, the interplay between individual and institution conditions PNS SPIs (Guimarães Pereira and Saltelli 2017).

Policy-relevant research, working with specific actors, is central to an approach called quantitative story-telling (QST) (Di Felice et al. 2021). The purpose of QST is to ensure that sufficient attention is given to how quantified metrics and indicators are produced and interpreted by specific actors, at a particular time, in a particular context for a specific purpose (Kovacic 2018; Voelker et al. 2019). QST is a particular form of participatory mixed methods to explore the performance of a system. QST follows a cycle of ‘semantic’ activities (engagement with stakeholders, identification and articulation of dominant framings and narratives) that are used to inform the ‘formal’ aspects of QST (quantified results from models or accounting frameworks). These results are deliberated on with attention to how different actors understand and respond to these metrics. The process of QST is relevant to SPI literatures, as the formal-analytic outcomes are appraised in terms of credibility, relevance and legitimacy (Giampietro et al. 2006; Allen and Giampietro 2016).

QST is designed to be consistent with a post-normal approach in several ways. First, through a focus on uncovering and discussing multiple problem frames at the start and end of the cycle to expose ontological uncertainties. Second, through a focus on ‘transdisciplinary teams’ interacting throughout the QST cycle. Third, most applications of QST have used a ‘societal metabolism accounting framework’ (Giampietro and Mayumi 2000) combining multiple simultaneous perspectives on flows and funds within both the biosphere and technosphere; across different decompositions of space and economic or demographic sectors. This multi-faceted and systemic approach to accounting includes externalities and was useful to enable an evaluation of sustainability. Thus, QST ensures the science part of a SPI is systemic and recognises multiple knowledge claims whilst the QST process highlights the importance of the interface to agree the problem frame before findings and solutions are debated. Policymakers are enrolled as part of the transdisciplinary team choosing the stories to follow and making sense of the quantified results. This paper does not describe the quantitative accounting aspects of QST, which can be found in existing literature (Ripoll Bosch et al. 2018; Krol et al. 2018; Giampietro et al. 2017). In short, the paper locates itself within the need for SPIs to advance sustainability solutions, explores the benefits of taking a PNS approach that recognises contestation and uncertainty, and the potential of the QST methodology to deliver a PNS-SPI. We consider how to address the complexity of multi-dimensional and uncertain aspects of steering the delivery of SDGs across multiple policy units. This challenge requires research with policy actors in transdisciplinary teams, rather than research on EU sustainable development policy-making for policy actors.

The paper has the following research objectives:

1. Highlight the benefits and challenges experienced working with EU level policy actors steering sustainable agricultural policy as part of the implementation of SDG2
2. Consider whether a PNS approach like QST can facilitate a systemic approach to sustainability.

The paper contributes to sustainability science through providing an individual case study on a PNS-SPI involving multiple policy institutions within the European Commission. The paper now describes the case, the policy context, the stages of QST and how the data were collected and analysed. The discussion highlights how the case compares to other empirical SPI papers. The paper therefore has relevance to those practising SPIs related to sustainability, even if the reader is not committed to using the QST approach specifically.

## Materials and methods

To examine the use of QST in a policy context, we implemented a QST cycle within the EU agricultural policy context (Matthews et al. 2017, 2019). This was the focus of one work-package within a broader multi-partner project called MAGIC,^1^ funded by the European Commission within the Horizon 2020 programme—this paper refers to the instance of implementing QST in an EU wide policy setting and focusses on the procedural findings with the accounting findings in a sister paper (Matthews et al. 2021).

### Focus

The QST approach was implemented in two cycles, each starting with a different (albeit connected) focal ‘story’. The first QST cycle (2017–18) focussed on “CAP aims to ensure European agricultural competitiveness in the world market and aims to deliver public goods, such as biodiversity conservation, water quality, and climate change mitigation. These aims are in opposition.” This focal story responded to the proposed revision of Common Agricultural Policy (CAP) objectives and pressure to integrate agricultural policy with delivery of climate and environmental objectives (Hodge et al. 2015). Calls for a systems approach to CAP (Kuhmonen 2018; Scown et al. 2019; Scown and Nicholas 2020) made the CAP an interesting focus for a PNS QST application. The CAP is a huge and complex policy, with a plethora of policy instruments nested underneath its objectives. The first cycle of QST was focussed on the outcomes of farming activities (as expressed through metrics generated from the Farm Accountancy Data Network^2^) and associations with material, financial and human inputs. It was not focussed on a particular policy instrument, farming sector or commodity, precisely to give a holistic overview.

The second QST cycle (2019–20) positioned itself in terms of how the CAP was becoming enrolled in delivery of the Sustainable Development Goals. The focus for the second cycle was “Sustainable agriculture is an important societal goal: it helps to support environmental policies as well as being a key component of SDG2. The European Commission claim CAP enables sustainable agriculture, supports environment policies, such as WFD and N2K, and supports progress to the SDGs. However, agricultural externalities harm European progress to achieve its sustainability goals both within and beyond Europe”. At the time of the second QST cycle, the EU’s SDG implementation had been criticised for being too focussed on encouraging development in non-EU States and neglecting its internal unsustainable trends (Szopik-Depczynska et al. 2018; Montéville and Kettunen 2019). Although the literature suggests focussing on the interactions of all 17 SDGs (Pradhan et al. 2017), we selected SDG2 (sustainable agriculture, nutrition, food security and ending hunger) as a more tractable focus, broadening the CAP-environment discussion of the first cycle into the wider food system and its impacts.

Although both cycles take a story and model simulation approach also used in participatory scenario analysis (Kok et al. 2015; Swart et al. 2004); the focus was on widening the frame by which past and current sustainability trends are understood, not on potential future actions.

### QST

The overall stages for each QST cycle described above are introduced in our project report (Matthews et al. 2020).

The cycle explains the sequence of methodological steps undertaken to derive the results presented below. 

1. Identify key themes relevant to policy: This stage draws on desktop analysis and primary data to identify the issues and ideas which are of interest to policy actors. This initiates the formation of ‘Transdisciplinary teams’ (comprising policy stakeholders as well as MAGIC scientists).
2. Decide what to represent in societal metabolism analysis: This stage develops a more specific shared understanding of what will be analysed and how to make the issues identified in stage 1 amenable to societal metabolism (flows of energy and material) accounting. This stage moves progressively from deciding higher-level priorities (the type and numbers of issues to be analysed), towards decisions on the specific aspects of systems that have to be represented that will shape the quantitative analysis (e.g. setting system boundaries, scales of analysis, useful indicators).
3. Compile data and carry out societal metabolism accounting: This stage sees the formalisation of the systems relevant to the themes identified in stage 1. The accounting framework is populated with quantitative data to become a quantified (and sometimes spatial) representation of the system. The first cycle used a societal metabolism approach focussed on associations between different Farm Accounting Data Network (FADN) indicators, whereas the second cycle used the full Multi-Scale Integrated Analysis of Societal and Ecosystem Metabolism (MuSIASEM) accounting methodology (Giampietro and Mayumi 2000).
4. Contextualise and present metrics: Metrics are generated that summarise the feasibility (within biophysical limits) and viability (within socio-economic limits) and desirability (distributional, burden sharing and acceptable outcomes) of the system as it is represented by the quantified data.
5. Discuss interpretations and implications: This stage sees the deliberative interpretation of the outputs of the QST analysis with policy actors. It is both a process of ‘closing the loop’ and the shaping of any further cycles—either with new stories or with alternative cases.

The aim is to complete the QST cycle in transdisciplinary teams to generate meaningful outputs that stimulate deliberations about the implications of these metrics for tackling sustainability challenges (Matthews et al. 2019).

### Data collection

At the start of the first QST cycle, eight policy actors from the European Commission and its agencies were interviewed regarding CAP evolution and its relationship to sustainable development (see Voelker et al. 2019). A content analysis was conducted of CAP in 2016–17 and these data were supplemented by field notes from a small cross-policy focus group with members of staff from different Directorates General (DG) —DG Agri, DG Energy, DG Environment and the Parliament’s Environment Committee held in May 2017. Deliberation over a choice of stories for QST was held via email and an online meeting with a further five policy actors from DG Agri in August 2017. Once societal metabolism analyses were completed and choices about which metrics to present and how to present them were made, a workshop was held in October 2018. The one and a half hour workshop was held in DG Agri meeting rooms in Brussels, facilitated by a contact in DG Agri and six DG Agri participants, some of whom had not previously been involved in MAGIC. The workshop consisted of a presentation introducing the project, the methodological approach, and the findings, ending with a series of questions for discussion.

To initiate the second QST cycle, we interviewed five new actors (two from DG Agri, two from an NGO and one from the Joint Research Centre) to fill in the gaps from our policy review of the EU implementation of SDGs during May 2019. Field notes from attending the Commission’s Green Week 2019 were also used for context. Three different workshops were held in November 2019, January 2020 and June 2020 (originally planned for March 2020 but delayed due to COVID-19). The first workshop was held in Brussels, at Scotland House, a venue independent from the official EU institutions, attended by fifteen participants from DG Devco, DG Clima, DG Agri, DG Research, EASME and a think tank. As with the first cycle, the one and a half hour workshop summarised the purpose of the research, the methodology and the findings. The second workshop was a ‘parliamentary breakfast’ hosted by a member of European Parliament, attended by twelve participants from the Commission and Parliament. The third workshop was a webinar with eight participants from the European Environment Agency and associated topic centre researchers. The second and third workshops used the same structure as the first workshop.

In both cycles, the last QST stage was designed to ask the policy actors to share their knowledge and expertise both during workshops and afterwards privately through feedback forms. A workshop report was provided to the participants with an invitation to follow up any points of interest with the MAGIC scientific team. To improve our understanding of how QST was experienced by the non-MAGIC participants, a colleague not involved in the workshops undertook four feedback interviews with participants from the Commission and agencies during April–June 2020.

The nature of the QST process means data were collected from a self-selecting sample of those motivated to engage with PNS and QST. Researchers also attempted to engage with people from other relevant Commission agencies, but were either unable to, as in the case of DG Sante, or had limited success, in the case of DG Env. Therefore, the results must be interpreted within these caveats. The interviews were recorded and transcribed. In all workshops, full field notes were taken by a dedicated researcher. The experience of the MAGIC science team was captured through internal meeting notes and research memos. The research received authorisation from the James Hutton Institute Research Ethics Committee.

### Data analysis

The transcripts, field notes, meeting notes and feedback form data were managed using the qualitative research database NVIVO 12 and labelled with case attributes to allow analysis by type of actor, policy domain and different settings (workshop or interview). Initial thematic coding illustrated what was learnt in the QST process for technical reporting (Matthews et al. 2020). Further focussed analysis, embracing theoretical multiplicity (Karpouzoglou et al. 2016), was used as illustrated in Table 1 below. The approach is a simplification of the multiple literatures introduced in the Introduction to provide three clear aspects with which to consider implementing a PNS approach to sustainability with EU policy actors.

**Table 1:** Analytical approach

Requirement	How these requirements were operationalised in QST
Systemic	Multi-dimensional metrics Multi-scale and multiple system perspectives Multiple policy domains
Post-normal	Uncertainty Unstructured problem finding Inclusive transdisciplinary teams Normative (political) approach
Science–policy interactions	Credible Relevant Legitimate Iterative

## Results

**Fig. 1:**
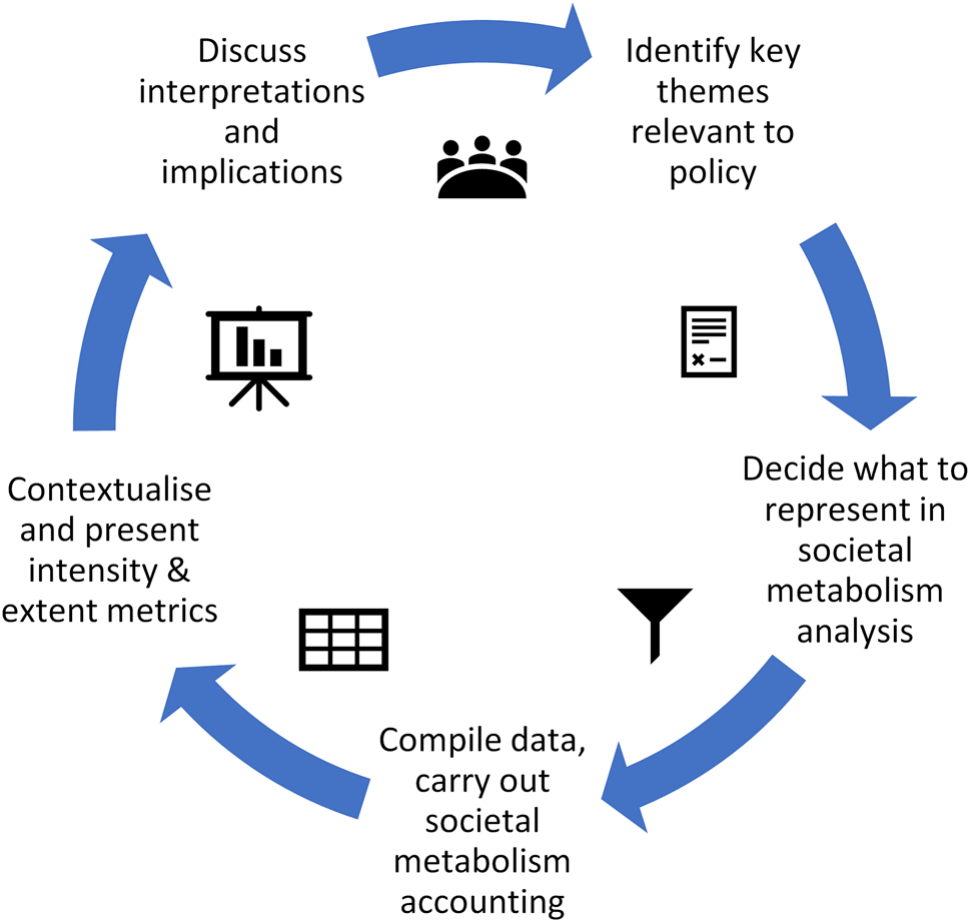
An overview of the quantitative story-telling (QST) cycle

The results cover the main learning points from the two cycles of QST (see Fig. 1) before addressing how QST supported policy for sustainability using the analytical framework (Table 1). Each section is a snapshot of the main themes summarising each aspect before the implications of the findings are discussed in Sect. 4.

### What did we learn about implementing QST?

Here we (the authors) set out the aspects that generated the most reflection about the practice of undertaking QST, discussed according to each stage of QST (Matthews et al. 2018, 2020).

1. Identify key themes relevant to policy: In both QST cycles, the scope was set by the project specification, but there were many potential themes. This stage proved difficult to achieve sufficient depth and breadth of analysis for relevant themes within the available time. More resources were spent in the first cycle on formal document analysis and interviews with relevant policy actors than on the second cycle. Policy actors were not asked to select the theme in the second cycle as this process extended the time taken on stage one without improving the quality of the discussion in stage five of the cycle. It was important to anticipate and iterate between stage 1 and stage 5 (the feedback stage) in terms of identifying themes relevant to identified actors. It was more important to invest in the last stage of the cycle than perfect the first stage.
2. Decide what to represent in societal metabolism analysis: This stage proved very important for the shared learning within the MAGIC researchers. We used the Eurostat Farm Accountancy Data Network (FADN) as the best dataset on which to base a pan-EU farm systems sustainability assessment but generated constraints regarding what data were available, at what resolution and decomposition by scale or sector (Matthews et al. 2019, 2021). Iteration occurred between stage 1 and stage 2, reappraising which themes were most promising given the data available. Stages 1 and 2 shape stage 3, regarding how thorough the accounting should be, and stages 4 and 5 regarding presenting the data. Using different societal metabolism methodologies in the first and second stages, to advance scientific knowledge, created extra work—using familiar methodologies in further iterations would make this stage more tractable. Feedback on the final choice of representations was shared with the policy actors during the first cycle. Discussion with policy actors at the end of the first cycle about what the FADN dataset could support helped guide the choices made in the second cycle.
3. Compile data and carry out societal metabolism accounting: Although the first QST cycle did not use a full MuSIASEM application, both QST cycles shared the challenge of taming the exponential growth in potential metrics being produced. There were many technical challenges, such as discontinuity in the FADN data available, limited access to the data, missing data values, particularly given it was the first experience of using MuSIASEM for most of the authors (Matthews et al. 2021). In both cycles, the application was in ‘diagnostic’ mode (analysis of current state) not ‘simulation’ mode. Running QST in simulation mode to discuss potential futures could be less confronting than discussing current policy choices. As with stage 1, it was important to balance the potential to deepen analyses with reserving resources for stages 4 and 5, as well as for further cycles.
4. Contextualise and present metrics: In both QST cycles, the societal metabolism applications produced metrics for environmental pressures (feasibility) and socio-economic pressures (viability), but desirability remained a product of deliberation reserved for stage 5. The environmental pressures were converted to spatial impacts in the second QST cycle. The main challenge for QST was considering which metrics to represent visually for deliberation in stage 5 (Cortes Arevalo et al. 2020). The final technical report contained complex Circos diagrams (Krzywinski et al. 2009); relationship maps and extent-intensity matrices (Matthews et al. 2020), but in both cycles, the presentations for policy actors focussed mainly on using bar charts, tables and two dimensional graphs, which were more familiar to the anticipated audience and required less explanation. Contextualising and explaining the ‘take-home’ message and the depth of analysis leading to this message took considerable effort. As with stage 2, stage 4 was a very important step in the QST cycle, as it required many choices about what to represent and how to represent the data. These processes are underreported in academic literature, yet are fundamental in shaping deliberations, particularly when there is limited time requiring a reduced selection of metrics. It was particularly difficult in the second cycle given the huge extent of data combinations possible. Therefore, this stage needs more time than one might expect.
5. Discuss interpretations and implications: In both cycles, we chose to use an existing seminar slot, recommended by policy actors in stage 1 of the first cycle as the timing and location would be convenient for the participants. However, this introduced time constraints (maximum 90 min) and perhaps generated expectations of a traditional ‘show and tell’ scientific presentation. The presentations asked about the political and bureaucratic configurations that block or enable transformation. In the second phase, we provided our own view of the implications before asking workshop participants whether they agreed with us. However, very little of the subsequent discussion addressed these questions. Provision of feedback forms, to enable people to express views privately also did not generate much insight. Even when discussing the results with environmental policy actors, most of the discussion was regarding the technicalities of societal metabolism and comparisons with lifecycle analysis. The innovation of ‘feedback’ interviews was extremely useful to get insights into how the policy actors experienced QST and we would recommend this as an important part of the QST cycle. This stage was both a way to close the QST cycle, but also the springboard for further QST cycles.

### How did the policy actors respond to the systemic nature of the QST?

**Fig. 2:**
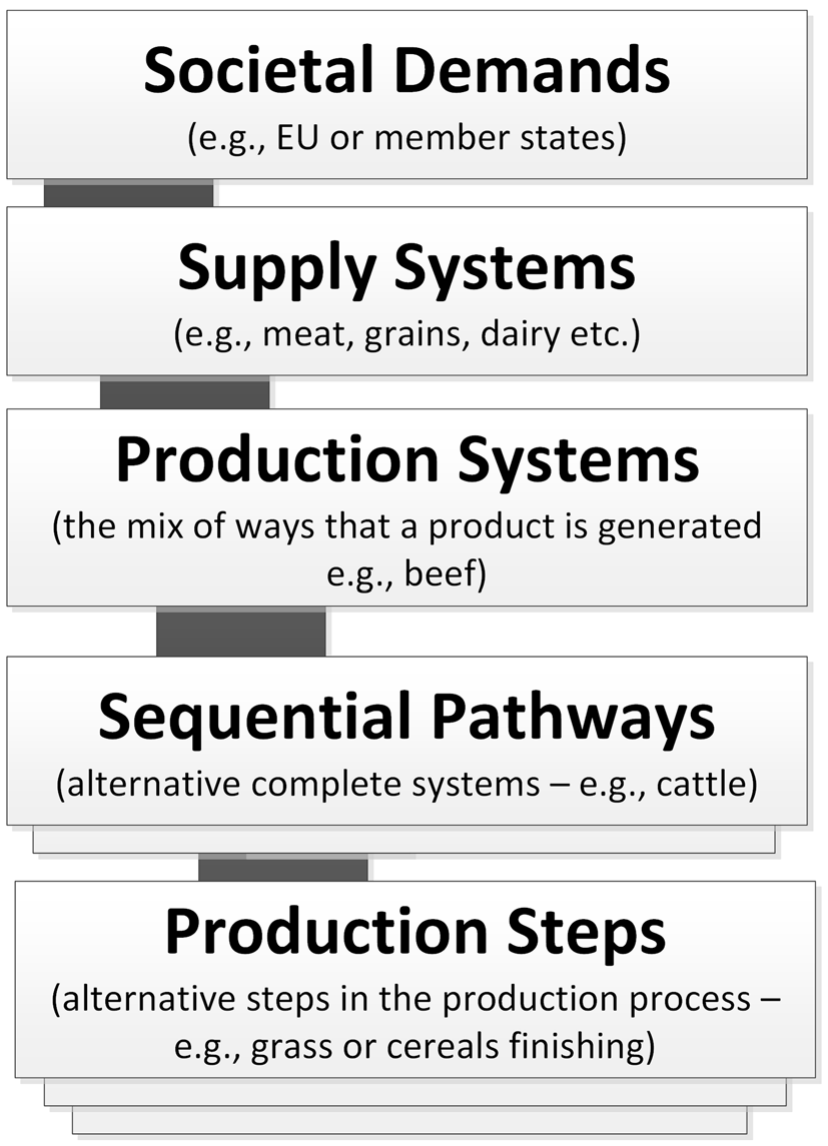
Different levels of the agri-food system

The first iteration of QST was less ambitious in coverage, focussed primarily on arguments around economic efficiency and environmental management by farm types and across member state regions. The second iteration of QST was more ambitious, extending beyond the farm gate into the agri-food supply system (see Fig. 2).

*Multi-dimensional metrics* (money and material) were used in both QST cycles. The metrics were presented by standard farm types (based on the European Farm Accountancy Data Network typology). The metrics addressed environmental pressures (and where possible, impacts) on soil, water quality, pollinators and water quantity and GHG emissions in the first cycle; and this was extended in the second cycle—for example, differentiating green and blue water. In the first cycle, metrics across EU28 were also produced for viability aspects, such as farm productivity, net farm income (before and after agricultural subsidy); and in the second cycle, more information, such as reliance on family labour and seasonal labour, was generated. The second cycle introduced data on nutritional demand and dietary choices. Central to societal metabolism analyses is the production of both extents (total hectarage, number of livestock, hours worked) and intensity (such as stocking rate per hectare, energy inputs per hour). In both cycles, visualisations were used to illustrate data in both extents and intensities, often on the same figure (e.g. soil erosion losses per ha, and total soil losses for each member state).

*Multi-scale analysis* was presented in both QST cycles. In the first cycle, environmental and social data were presented by member state and NUTS2 regions and by different farm type; and intensity metrics presented by hectare, hour or euro. In the first cycle, change over time was indicated, but the second phase tended to produce static data for a single year. In both cycles, EU commodity imports and exports were explored; with the second phase introducing the dependence of EU agricultural commodities on imported materials and labour, illustrating the difficulties of reducing inputs from beyond the EU.

*Multiple policy domains* were involved in both cycles, with participants from different DGs (see Sect. 2.3) with interests in climate, development, agriculture and the environment. The primary policy focus was the CAP, but the results for water, soil and pollinators were relevant to the environmental fiche; and GHG results relevant to climate action. In the second cycle, CAP was a nexus for multiple aspects of the SDGs, but the data were not presented against policy objectives nor individual policy instruments. Instead, the data were presented against the four main components of SDG2, to locate CAP and its sister policies within a wider sustainability framing.

Overall, there was a mixed response to the systemic nature of the QST results. Whilst most policy actors were interested in the different aspects of the system, the societal metabolism accounting was unfamiliar and difficult to understand (see Sect. 3.3). It was also time-consuming to explain the extent and intensity metrics. Participants were given printed slides at the workshops, which allowed them to flick between guidance on how to read the diagrams and the results. The discussion may have been impeded by the lack of understanding, although there were moments when participants suddenly recognised the implications—such as the focus on intensity of GHG emissions in places like the Netherlands neglected the cumulative effect of low GHG emissions over large extents in places like France. There was some resistance to the implication that EU agriculture is not sustainable from the DG participants, although broadly accepted by the NGOs, agency and MEP participants. Many DG Agri participants wanted to focus on how the CAP was evolving to be more focussed on environmental and social protection. Whilst the systemic presentation was often seen as intriguing, for most actors, it was not seen as particularly relevant (see Sect. 3.3).

### How did the policy actors respond to the post-normal nature of the QST?

The interviews and workshops did not use the language of post-normal science explicitly, but the main tenets of PNS were addressed in the design and content of the QST cycles.

*Uncertainty* analysis is extremely important to PNS approaches, as it reinforces the philosophy of scientific bounded rationality and knowledge as always in the making. However, in both cycles, there was limited time to discuss uncertainty and sensitivity analyses. The metrics were intended to spark discussion and were presented as partial and illustrative. Participants seemed to understand that we were discussing a partial view on complex and adaptive socio-ecological systems.

*Unstructured problem finding*, ensuring that the nature of the problems is understood and agreed on, is central to PNS. Whilst there is a growing academic critique of CAP and the EU’s approach to agri-food sustainability, we wanted to explore how policy actors understood these problems before discussing how to transform the system using policy levers. Problem framing is important when policy levers can worsen the systemic problems through not accounting for wider consequences. It proved very difficult to get policy actors to discuss systemic problems (see Sect. 3.1). Instead, the deliberation focussed on how policy actors were making progress in resolving the problems. In other words, where critiques were acknowledged, they were recast as being resolved by technical solutions. The feedback interviews revealed that the participants had expected the scientists to present solutions and confirmed that asking policy actors to work with scientists to develop a shared understanding of the problem was highly unusual. Although we emphasised knowledge co-creation in the recruitment material, we realised that individuals attended the workshop in passive knowledge recipient mode. In the future iterations, building capacity around knowledge co-creation is needed.

*Inclusive transdisciplinary teams* underlie QST. We hoped to engage policy actors as co-researchers throughout multiple cycles. Whilst we did engage some actors throughout both cycles (see Sect. 2.3), there was much less knowledge co-creation than planned. This was partly due to their limited availability and different priorities—policy actors were navigating changing policy priorities, from the past EU sustainability strategy to the delivery of the SDGs, working on the ex-ante impact evaluation of the proposed new CAP and delivering the Juncker Commission. Being relevant (see Sect. 3.4) meant targeting extremely busy and focussed actors. The ongoing scientific learning within the research team regarding how to undertake each step of the QST cycle meant we informed rather than actively engaged the policy actors regarding choices in stages 2, 3 and 4 of both cycles. In the first cycle, a member of DG Agri offered to champion the project with their colleagues, but they left, and we did not find a replacement. Engaging beyond DG Agri in the second cycle took time to research multiple DGs and other units within the Commission. However, providing the opportunity for interaction between DGs (beyond the formal Inter-Service Steering Groups convened for specific policy processes) was most appreciated according to feedback sheets and interviews. Thus, whilst the transdisciplinary teams were not as active as originally hoped, we did engage a range of EU level policy actors in both cycles, which we believe was a partial success.

*A normative approach* is the final tenet of PNS, recognising how values and politics are integral to how science is understood. QST is not about value-neutral presentation of facts but acknowledges the values associated with generating and interpreting the societal metabolism metrics. This was probably the most dissonant aspect in the three workshops. The field notes suggest that the general atmosphere was polite and reserved, with the discussion focussed on technical matters of professional expertise rather than the political implications or personal passions. There were exceptions when individuals grew animated in the workshops. The interviews, feedback forms, and follow-up emails revealed some normative views, but these varied from person to person. Some individuals still focussed on what they did and how they did it, with little commentary on why it mattered or how it felt. There was much less dissonance with the parliamentarians, who were willing to acknowledge how the implications might affect them. We debated many ways of trying to elicit values to complement the stage five discussions, such as using a multi-criteria approach. However, given some participants struggled to understand the method, and debated the metrics, such structured exercises were inappropriate. Ideally, there would be multiple interactions to split up the presentation of the results from debating the implications for their roles and responsibilities. However, time constraints meant both were addressed in the single workshop.

Overall, there was a mixed response to the PNS aspects of QST. Taking a PNS approach meant our workshops were very different to previous SPIs. There was some resistance to structuring the problem, with the actors either accepting that there were major problems and expecting us to have solutions; or arguing that the Commission was already working on suitable solutions, so they did not want to discuss if the systemic nature of problems was fully understood. Policy actors are heterogeneous. There were some engaged actors who were positive about the potential for QST to assist their work. Feedback interviews revealed that there were some actors who wanted more engagement with the researchers. We had followed up requests for information, but as we were unfamiliar with the individuals, we didn’t understand they were signalling a desire for further active science–policy interactions.

### How did policy actors respond to the science–policy interface aspect of the QST?

Here we briefly reflect on how QST relates to the four tenets of positive SPIs.

*Credibility* The quantification in the QST cycle used societal metabolism. This systemic accounting process is not a typical approach and its ontology is opposed to many of the conventional policy assessment methodologies such as cost–benefit analyses (Giampietro et al. 2013; Saltelli and Giampietro 2017b). It was difficult to explain the methods and how to interpret the findings in a short time and participants may not have fully understood the purpose of societal metabolism. Lack of familiarity could have reduced participants’ ability to judge the quality of the evidence. Given the constraints on overall workshop length, the presentations were highly selective and provided a very partial perspective. In both cycles, participants were unused to having ‘draft’ results presented to them for discussion and improvement. Together, these aspects may have undermined the credibility of the results, particularly if judged as evidence for policymaking rather than starting points for co-creating pathways for improved policy. It is unlikely that participants would directly challenge the research team’s credibility, but the questions about methodology could be read as an oblique test of our expertise (Waylen et al. 2023).

*Relevance* The authors explicitly selected EU policies as the focus for the QST to test how QST might be used to support policy cycles (Matthews et al. 2020). Participants found it useful to focus on competitiveness as this was the topic of the European Semester (framework for coordinating economic analyses). Given feedback from the first phase interviews, we switched from the original language of Water–Energy–Food Nexus to language of the CAP objectives; and participants appreciated the workshop in 2018 having been informed by prior interactions with DG Agri. By the second cycle, the Commission were being challenged for more detail on their approach to the SDGs; whilst the Green Deal (European Commission 2019) and the Farm-to-Fork Strategy (European Commission 2020) were under development. The QST cycles had to support a fast-moving policy landscape. This situation was challenging given that the specific focus, language and framing of these policies were often not visible and had to be accessed through interviews and informed guesswork at the start of each cycle. The holistic approach in both QST cycles meant that the analyses lacked relevance to individual policy actors with responsibilities. Indeed, some wanted us to explore technical aspects of production steps or sequential pathways (Fig. 2), which related to specific policy instruments (e.g. support for organic farming). Towards the end of the project, contrasting the quantified results from societal metabolism analysis with the results presented under the Common Monitoring and Evaluation Framework for CAP was suggested as one solution.

*Legitimacy* QST aims to make choices explicit at each stage of the cycle. It was difficult to fully explain the quantification process and impossible to present all the quantified data in the time-limited workshops, making the QST process not fully transparent. The workshops were designed to ensure inclusive participation and to encourage a range of values to be expressed. The range of participants from across different DGs and with different roles was appreciated by participants, but the limited discussion did not really improve mutual understanding between DGs or between the researchers and the rest of the ‘transdisciplinary teams’. The presentation of the results as partial positioned all actors as knowledgeable, rather than researchers imparting knowledge to the policy actors, but this did not work particularly well. The interviews helped us to understand policy processes but did not involve discussion of the QST results. Whilst more one-to-one interactions at stage five would improve mutual understanding, it would fail to build collective mutual understanding between the DGs. The authors felt a shared problem framing within the Commission is necessary to support SDG2.

*Iterativity* QST is evolutionary, building on reflection at each step to deepen relationships and foster improved understanding. It is not simple repetition but extending contextualised knowledge production. Between the first and second QST cycle, we faced a choice about whether to continue with the same narrative (CAP’s tension between competitiveness and public goods) or to broaden the focus. The former would have allowed further iterations. It may have been more relevant to one or two actors, but it diverted the focus away from a systemic approach to sustainability into a specific aspect on production steps. Instead, we chose to extend the QST to look more explicitly at how CAP was enrolled in delivering of the UN SDGs, through coherence with other policies (see Sect. 2.1). This allowed us to build on the first cycle but to bring in new institutional settings, practices and actors beyond DG Agri. This amplified the learning for the researchers and provided a greater network of policy actors with whom to learn. However, it meant there was less time to consolidate learning about specific policies and actors and perhaps less ability to improve our credibility or the quality of these relationships. Originally, we planned to run both QSTs in simulation mode as well as diagnostic mode, to consider possible future trajectories. This is another set of choices in how and when to iterate. We chose to focus on analysis of the current systemic metrics, rather than hypothetical alternatives, as we felt this was more relevant and more credible. However, comparing our experiences with others using QST in the project (Krol et al. 2018; Cabello et al. 2021; Di Felice et al. 2021) albeit with different actors and topics, simulations may prove better for building relationships as they do not entail any evaluation of past or current policy performance.

As with the responses to the systemic and PNS aspects of QST, our experiences with QST as a SPI were also mixed. Our experiences suggest that legitimacy was somewhat in tension with credibility, as trying to co-construct knowledge through a new methodology in a collective setting was very challenging. These observations reinforce the need for iterativity to build familiarity with the methodology and to build relationships of trust where values and positions can be expressed. In the future, more frequent meetings are needed. We were too ambitious in the shift between first and second cycles, which impacted on our ability to be relevant in the right way, with the right actors, at the right time. However, we believe QST could be a very useful approach for future SPIs, building on the lessons we have learnt.

### Supporting policy for sustainability

Policy coherence is central to the delivery of the UN and EU 2030 agendas, requiring both a broad and deep understanding of how objectives, instruments and implementation practices can be supported across policy domains. However, it was very difficult to assess how policy coherence for the SDGs was practised within the Commission. During the first stage of the second cycle, considerable time and effort were expended on trying to trace how the SDGs were being implemented, given there was limited public information on how the lists of policies and strategies would be combined.

We also struggled to identify how different parts of the Commission were involved. Whilst it was clear that the Secretariat General’s department was responsible for the overall implementation of the SDGs, we were unable to get more detail on these processes. Approaches to the Secretariat General’s office were redirected to the DGs, yet individual DG staff were responsible for aspects of a single policy. We were aware of, but unable to access, Inter-Service Steering Groups such as the group consulted on the proposed new CAP objectives. It would have been ideal to use QST with such a collective. Instead, we involved some actors from the CAP inter-service group, but most actors were very focussed on the implications for their own role within a specific policy domain. The fact that cross-DG attendance at seminars was welcome but unusual suggests that these inter-DG relationships are still nascent and not a routine part of the job.

In fact, the MEPs and NGO actors were the most interested in a systemic overview, yet these were not the original targets for the creation of our ‘transdisciplinary teams’. The approach was also more relevant for agencies, such as the European Environment Agency or Eurostat. However, to focus on these other institutions does not address the need to reframe EU polices to address the transformation required to meet the UN and EU 2030 agenda. These observations reinforce the challenge of using QST to address SDGs with Commission actors, rather than using QST to address a particular issue within a DG and policy domain. Given the importance of iteration, further cycles of QST could have been used to build up through the system levels (see Fig. 2) and beyond the original starting DG, but more slowly than we attempted. The process of using QST for SDG2 was beginning to mature when the project ended. We compare our learning points with relevant published experiences, to discuss the potential of QST to support policy for sustainability.

## Discussion: trade-offs and governance challenges

This paper adds to the growing literature on how SPIs involve choices and trade-offs. In particular, there is a wealth of literature reflecting on transdisciplinary research on sustainability (Schneider and Buser 2018) and the SDGs (Schneider et al. 2019); as well as advice on how to practise PNS appropriately (Kønig et al. 2017). Our findings acknowledge where—for lack of resources, knowledge or foresight—the implementation of our QST cycles did not meet these exacting requirements. For example, we did not have sufficient ongoing relationship building resources (Bergmann et al. 2021) and struggled to have the intense involvement required (Brandt et al. 2013). Therefore, our case reflects on implementing QST as a PNS SPI tackling pan-EU policy and we highlight areas of struggle as well as success and our reflection may be useful to other researchers embarking on similar processes.

Using a less complex methodology that did not address multiple simultaneous dimensions might have made it easier for non-scientists to engage with the metrics and their implications. However, the choice to use societal metabolism accounting within our PNS SPI responds to critiques that many analyses of SDGs have limited systemic analyses (Miola et al. 2019). Spending less time on describing and implementing the accounting may have allowed more frequent QST iterations and improved the SPI practice (Sarkki et al. 2015). Therefore, our case suggested a trade-off between highlighting all dimensions of sustainability wicked problems, and the tractability of using complex systems methodology in transdisciplinary teams where there was limited social capital and low familiarity with societal metabolism accounting. We maintain that it is important to aspire to a truly systemic approach within SPIs but with hindsight, attempting to tackle SDG2 in the 2nd cycle took resources away from relationship and capacity building processes within DG Agri.

Trying to go inside the ‘policy-making black box’ (Gossling et al. 2016) was ambitious to attempt QST in a new institutional setting where we had no existing networks or prior experience to help us negotiate knowledge co-production. For example, we read policy-makers’ request to focus on organics as deflection to an incremental focus on technical solutions, avoiding a discussion on the systemic problems with CAP (Kuhmonen 2018). With hindsight, it might have been an attempt to bolster the position for agro-ecology in the new Commission and increase the environmental ambition of the Farm-to-Fork Strategy. Likewise, we discovered it is important to keep contacting policy actors despite their limited interest or opportunity to engage. Approaches like QST are emergent and co-produced by the specific time and context—they are therefore more difficult to predict, plan and control (Chilvers and Kearnes 2019) and require continuous adaptation (Oliver et al. 2021).

However, attention to the SPI design and good practice is necessary but not sufficient. We identified the policy windows (Rose et al. 2017); but improved design and greater determination could not address the problematic governance across silos (Ruddy and Hilty 2008; Candel and Biesbroek 2016). Our experience—that SDGs were of interest to everyone, but the responsibility of few—was also highlighted by others (European Parliament 2019; Montéville and Kettunen 2019). This is a common governance challenge, whereby the more holistic the issues, the more diffuse, contested and invisible the processes of accountability become (Bevir 2011; Kraft and Wolf 2018). It is difficult to be relevant when few have a remit for tackling wicked problems [also highlighted when working with Swedish policy-makers (Höjer et al. 2011)]. Having a policy entrepreneur to advocate for change would have helped identify systemic champions to whom the societal metabolism metrics were most relevant (Timmermans et al. 2014).

Many other SPIs use future methodologies to explore plausible but hypothetical pathways to sustainability to support policy development and implementation (Höjer et al. 2011; Carlsson et al. 2015). Indeed sustainability science often uses science to tackle urgent societal challenges; with SPIs co-producing solutions (Bergmann et al. 2021; Miller et al. 2013). However, our PNS approach asked policy-makers to (re)consider how current policy choices were generating unsustainable trends, across multiple domains and spatial scales throughout the EU. Far from offering solutions, QST tries to reframe the debate away from incremental progress towards acceptance that more radical change was needed (Saltelli and Giampietro 2017a). However, starting with potential solutions, whilst somewhat contrary to PNS philosophy, could have built collective capacity in transdisciplinary teams, which could have been harnessed to later acknowledge and address wicked problems (Termeer et al. 2015).

Positive process design cannot always defuse unpalatable content. The QST results (Matthews et al. 2017, 2019) that found how the current approach by the EU is unlikely to achieve SDG2, is echoed in official reports (Eurostat 2019; European Environment Agency 2020). Likewise, the finding that the current framing of the problem is too narrow to understand and respond to the sustainability challenges, is also found elsewhere (Scown and Nicholas 2020). It is tempting to blame our challenges faced on the post-political nature of the European Commission (Wilson and Swyngedouw 2014), whereby solutions are framed within a neoliberal incremental approach to sustainability. This would imply that the SDGs can be achieved by existing policies implemented more efficiently and more effectively, but without challenging the status quo. It is difficult to balance a critique of the status quo with a more positive solutions focus; given true transformation often requires wholescale systems change, not incremental policy improvements (Chan et al. 2020; Dorninger et al. 2020). Our experiences with QST may have been difficult as we were asking for a deep transformation of the socio-ecological system, which challenges the status quo (Ojha et al. 2022). Non-state actors may be more willing to discuss the politics of the problems and embrace the challenge (Linnenluecke et al. 2017).

QST asked policy actors to go beyond commenting on the credibility and relevance of system knowledge (Schneider and Buser 2018), to reveal the unstated play of the policy-making game, rather than the formally recorded rules of the game. Our process emphasised the political dimensions and choices involved in the framing of societal challenges. Highlight the politics risks delegitimising the presentation of policy-making as rational-, linear-, and evidence-based (Cairney 2017, 2022). If our results are ‘uncomfortable knowledge’ (Rayner 2012) then QST becomes an uncomfortable process. Being willing to participate in an uncomfortable process requires commitment by these policy actors when there is little institutional support for doing so (Maas et al. 2022). It is possible therefore to reframe our limited engagements, particularly with those actors who stuck with the process over 3 years, as quite a success.

Despite many warnings that attempting to do QST on SDGs within the Commission DGs was impossible, we have achieved some small steps forward. The quantified findings provide a resource, with many others, to help shift how the Commission addresses SDG2, for example to reframe the Farm Accountancy Data Network to the Farm Sustainability Data Network (European Commission 2021) and to address impacts on non-EU biodiversity when considering the sustainability of intensive livestock rearing (Cadillo-Benalcazar et al. 2020). Echoing other findings (Guimarães Pereira and Saltelli 2017), it is clear that some institutional changes with the Commission are required that make space for pluralities of science practices and encourage greater reflexivity by both scientists and policy actors. We need engaged policy actors in the creation and not only consumption of sustainability knowledge (van Hulst and Yanow 2016); and actors willing to accept the breadth and scale of wicked problems.

## Conclusion: using quantitative story-telling with EU policy actors

This paper summarises the learning points derived from using two cycles of a post-normal science (PNS) methodology, quantitative story-telling, with EU policy actors. Each QST cycle covered issue framing, running societal metabolism analyses, selecting and presenting metrics and deliberating over what the metrics mean for policy actors (see Sect. 2.2). The first cycle focussed on a potential tension between EU agricultural competitiveness and provision of public goods; and the second cycle on delivery of SDG2 (zero hunger). Operationalising QST with busy EU policy actors and using science–policy interfaces (SPIs) with PNS systemic approaches was challenging (see Sects. 3.2, 3.3, 3.4). We achieved a partial success in stimulating new thinking about policies for SDG2 by Europe (see Sect. 3.5).

Some shortcomings could be overcome, by having more resources, experience and familiarity with the context and actors—to better prepare participants about what to expect from PNS approaches; build capacity in understanding societal metabolism and visualisations; and hold more frequent iterations, building on prior QST cycles. However, other shortcomings remain more challenging—such as trying to move from incremental business as usual improvements towards a more fundamental transformation and identifying the actors to whom such a proposition is relevant. Like all SPIs, QST requires a strongly relational approach, which asks for policy actors’ time, energy and trust. Unlike many SPIs, this is required whilst challenging many of the formal institutions on which the Commission’s authority is based.

Whether or not future academics adopt the societal metabolism accounting approach (MuSIASEM), we propose the QST cycle as a useful framework for PNS SPIs. Although challenging to implement, QST is rare in balancing multiple perspectives on problem framing with deliberation over integrated, holistic and cross-scale quantitative assessment of current situations. Explicit attention to problem framing before seeking solutions is fundamental to a PNS and deep sustainability. Potentially, the challenging the problem framing of current policies could be combined with other solution- or future-orientated SPI approaches that are more attractive to policy actors.

As existing SPI literature highlights, QST should be guided by a policy entrepreneur and seek inputs from a diverse ‘transdisciplinary team’. Users should expect to expend significant time on decisions around selecting and visualising metrics, combined with additional time to reflect on the effects of these decisions. Using QST requires not only a strong academic team with multiple scientific specialisms but also wider transdisciplinary skills, such as relationship maintenance, political sensitivity, determination, and confidence.

However, using QST also has implications for non-academic members of QST transdisciplinary teams (Stokols 2006). QST is a means to an end—a way to highlight the need to act more urgently on sustainability problems—and researchers cannot enact sustainability transformations alone. Therefore, QST requires actors able and willing to enact change, beyond incremental technical improvements to policy. Individual policy actors therefore need to step out of their bureaucratic remits and explore challenging and uncertain topics. Although the current climate, biodiversity, and COVID-19 crises require transformation to resolve them in the longer term, they are also immediate priorities for policy actors, further reducing their capacity and appetite for more apparently esoteric analyses.

We offer this paper as a contribution to the growing body of literature that reflects honestly on implementing SPIs in practice. We document what has been learnt when using a PNS approach at a pan-EU scale. Our observations add to those from other QST research (Cabello et al. 2021; Di Felice et al. 2021) and we would encourage further research using QST as a PNS SPI approach. It would be interesting to see if others fare better when using QST for SDGs; or if QST has more traction with other types of actors (activists, citizens) or when focussed on a specific policy. Our experience also adds to wider scholarship on taking a critical approach to SPIs. Such research will strengthen a post-normal response to sustainability transformation that is sorely needed.

## Data Availability

Many of the datasets generated by the H2020 MAGIC project used for this article are available in the Zenodo repository: https://zenodo.org/communities/magic/search?page=1&size=20&type=dataset. However, some of the datasets analysed for this article (e.g. the interview and seminar transcripts) are not publicly available due to participants being promised anonymity and confidentiality as part of their informed consent. When all information that could identify the individual or their organisation is removed from these transcripts, they no longer have much value as research data, as the information is context specific.

## Footnotes

1 The H2020 MAGIC project (Moving Towards Adaptive Governance in Complexity: Informing Nexus Security) is described and reported at https://cordis.europa.eu/project/id/689669

2 https://ec.europa.eu/info/food-farming-fisheries/farming/facts-and-figures/farms-farming-and-innovation/structures-and-economics/economics/fadn_en
